# Invited Discussion: The Battle for the Truth: Social Media Versus Critical Appraisal

**DOI:** 10.1177/22925503231162026

**Published:** 2023-03-13

**Authors:** Jennifer L. Giuffre, Andrew Simpson, Carolyn Levis

**Affiliations:** 1 Department of Surgery, University of Manitoba, Winnipeg, Manitoba, Canada; 2 Department of Surgery, 6221 Western University, London, Ontario, Canada; 3 Department of Surgery, 3710 McMaster University, Hamilton, Ontario, Canada

We read with interest a recent editorial in Plastic Surgery entitled “The Battle for the Truth: Social Media versus Critical Appraisal.”^1^ Social media has become pervasive. In the United States, the proportion of adults using social media has increased from 8% in 2005 to 72% in 2014.^2^ According to a 2019 EMarketer report, 90.4% of Millennials, 77.5% of Generation X, and 48.2% of Baby Boomers were using social media in the United States.^3^ Patients, students, trainees, and established surgeons are increasingly turning to social media for information. Although one would argue that through various platforms, social media is an important channel for dissemination of information to colleagues and patients, the counterargument is that the quality of the information may be unreliable and questionable.^4^ With the pervasiveness of social media, users must exercise critical appraisal of the posted online information to avoid being misinformed or placed into the “internet cave” with illusory truths.

The Canadian Society of Plastic Surgeons (CSPS) recognizes the importance of distributing reliable information and truths; therefore, has taken the initiative to develop a social media presence through Facebook and Instagram which will: present factual and reliable information regarding the history of Plastic Surgery, and the field of Plastic Surgery; discuss popular topics, dispel myths, highlight past landmark and current research papers and feature various Plastic Surgery procedures and surgeons and make announcements regarding the CSPS. When used sensibly, social media platforms can promote professional development, patient education and public health, however, when used carelessly, it risks the distribution of poor-quality information, breaches in patient privacy, violation of personal-professional boundaries, damage to professional reputation, and the potential for legal consequences. The CSPS's social media goals are to improve the quality of care through education, outreach, and advocacy to the CSPS members, trainees, and the public about Plastic Surgery.

Instagram and Facebook: cspscanadianplasticsurgeons, Canadian Society of Plastic Surgeons.
